# Physical health monitoring in dementia and associations with ethnicity: a descriptive study using electronic health records

**DOI:** 10.3399/bjgpopen20X101080

**Published:** 2020-09-23

**Authors:** Cini Bhanu, Mary Elizabeth Jones, Kate Walters, Irene Petersen, Jill Manthorpe, Rosalind Raine, Naaheed Mukadam, Claudia Cooper

**Affiliations:** 1 Department of Primary Care and Population Health, University College London Research, London, UK; 2 NIHR Health and Social Care Workforce Research Unit, King’s College London, London, UK; 3 Epidemiology and Public Health, University College London, London, UK; 4 Division of Psychiatry, University College London, London, UK

**Keywords:** dementia, large database research, inequalities, ethnic groups, primary health care

## Abstract

**Background:**

Good physical health monitoring can increase quality of life for people with dementia, but the monitoring may vary and ethnic inequalities may exist.

**Aim:**

To investigate UK primary care routine physical health monitoring for people with dementia by: (a) ethnic groups, and (b) comorbidity status.

**Design & setting:**

A retrospective cohort study was undertaken using electronic primary care records in the UK.

**Method:**

Physical health monitoring was compared in people with dementia from white, black, and Asian ethnic groups and compared those with ≥1 comorbidity versus no comorbidity, from 1 April 2015 to 31 March 2016. Using the *Dementia* *:* *Good Care Planning* framework and expert consensus, good care was defined as receiving, within 1 year: a dementia review; a blood pressure (BP) check (at least one); a GP consultation (at least one); a weight and/or body mass index (BMI) recording (at least one); and an influenza vaccination.

**Results:**

Of 20 821 people with dementia, 68% received a dementia review, 80% at least one BP recording, 97% at least one GP contact, 48% a weight and/or BMI recording, and 81% an influenza vaccination in 1 year. Compared with white people, black people were 23% less likely and Asian people 16% less likely to have weight recorded (adjusted incidence rate ratio [IRR] = 0.77, 95% confidence interval [CI] = 0.60 to 0.98/0.84, 0.71 to 1.00). People without comorbidities were less likely to have weight recorded (adjusted IRR = 0.74, 95% CI = 0.69 to 0.79) and BP monitored (adjusted IRR = 0.71, 95% CI = 0.68 to 0.75).

**Conclusion:**

Ethnic group was not associated with differences in physical health monitoring, other than weight monitoring. Comorbidity status was associated with weight and BP monitoring. Physical health monitoring in dementia, in particular nutrition, requires improvement.

## How this fits in

Good physical health supports quality of life for people living with dementia. Routine physical health monitoring in general practice can support this and should be accessible to all. This is the first study to explore routine GP physical health monitoring and ethnic group in people living with dementia. Findings can inform policies, promoting access to good post-diagnostic support.

## Introduction

Around 850 000 people live with dementia in the UK.^1^ By 2040, this number is likely to increase by 57%.^2^ People with dementia have high rates of physical morbidities (commonly hypertension and diabetes) that are primarily managed in general practice.^3^ Reducing physical morbidity improves quality of life for people with dementia^4^ and may slow cognitive decline.^5,6^

The Dementia: Good Care Planning framework recommended an annual GP review as a minimum standard of care, as a Quality Outcomes Framework (QOF) measure in 2015.^7^ Routine physical health monitoring should include tailored management and prevention of comorbidities, with medication, nutrition, and hydration reviews.^7^

UK policies prioritise fair access to health care,^8^ but inequalities exist. In the UK, people from black ethnic groups living with dementia are less likely to be diagnosed, and people from Asian backgrounds less likely to receive symptomatic treatments post-diagnosis, compared with people from white ethnic groups.^9,10^ Rates of physical morbidities are higher among people from black and Asian backgrounds compared with the white population,^9,11^ so it would be concerning if health inequalities extend to preventive care for physical disorders.

The study aimed to examine routine physical health monitoring for people with dementia in UK primary care and investigate whether this differs between white, black, and Asian ethnic groups as the main objective. As a secondary objective, the study investigated physical health monitoring for the overall population by comorbidity status. Informed by guidelines^7^ and expert consensus, routine physical health monitoring was defined for the study (see Outcome and patient characteristics for the definition).

## Method

This cohort study used electronic primary care records from UK GP practices contributing to The Health Improvement Network (THIN) database,^12^ which is broadly representative of the UK population. At the time of data collection, THIN included 744 general practices and 15.6 million patients in 2016. In the UK, most physical health monitoring of people with dementia happens in primary care.^13^ Clinical data are coded using Read codes.^14^ THIN captures demographic information on: sex, ethnic group, birth year, and patient-level Townsend score (an area-based measure of social deprivation).^15,16^

### Study population

Individuals included were aged 50–105 years contributing to THIN between 1 April 2015 and 31 March 2016 to coincide with the QOF recording year. Individuals with dementia were defined by a Read code indicating a dementia diagnosis or an anti-dementia drug prescription (cholinesterase inhibitors or memantine), as in previous studies.^9,10^ Individuals who were registered with a GP practice in the relevant period and had received a dementia diagnosis or been prescribed anti-dementia medication prior to that date (that is, had a dementia diagnosis for the whole study period) were included. All GP practices met standard criteria for acceptable mortality reporting and computer usage.^17,18^

### Outcome and patient characteristics

Good routine physical health care was defined as, over 1 year, a record of the following being received: a dementia review (at least one recorded); a BP measurement (at least one recorded); a GP consultation (at least one, in person or via telephone); weight and/or BMI recorded (at least one); and an influenza vaccination.

Ethnic groups were catergorised based on Office for National Statistics classification:^9^ white (British or other white background); Asian (Indian, Pakistani, Bangladeshi, or other Asian background); black (African, Caribbean, or other black background); and mixed or other ethnic groups (mixed, Chinese, or Arab).^9^ Individuals from mixed or other ethnic groups were excluded from analyses owing to small numbers (<0.10% cohort). For comorbidity status, people were identified with a diagnosis of hypertension, myocardial infarction (MI), stroke, diabetes, or chronic kidney disease (CKD). Covariates were age, sex, Townsend score, comorbidities, and prescribing index (a valid additional measure of comorbidity^19^). Age was grouped in 10-year categories; the last category was 90–105 years owing to few individuals being aged >100 years.

### Analyses

Multivariable Poisson regression was used to investigate the relationship between ethnic group and the outcomes. Models were adjusted for age, sex, Townsend deprivation, comorbidities, and prescribing index. IRRs were calculated for outcomes comparing black and Asian ethnic groups with the white ethnic group. Comorbidities were also stratified comparing the cohort with and without comorbidity.

Characteristics of individuals were compared with and without the ethnic group recorded, and complete case analyses were conducted for those with a record of ethnic group and Townsend score. Missing ethnic group data were then imputed using multiple imputation by chained equations.^20^ Compared with complete case analysis, multiple imputation can greatly strengthen results by collating all the information available from individuals with observed data.^21^ This way, information was used from the full dataset in the analysis to provide more precise estimates of the outcomes (see Supplementary Box 1). The multiple imputation results presented in this article are considered to be the primary results. They were consistent with the complete case analysis. Stata (version 15.1) was used.

## Results

### Included individuals

Of the 20 821 individuals aged 50–105 years in the study, 10 570 (50.8%) had their ethnic group recorded. Of 10 570 individuals with a recorded ethnic group, 96.6% (10 215) were recorded as from a white ethnic group, 2.1% (221) from an Asian ethnic group and 1.3% (134) from a black ethnic group. After multiple imputation of ethnic group, 96.6% were from a white ethnic group, 2.1% from an Asian ethnic group and 1.2% from a black ethnic group (Table 1).

**Table 1.** Participant characteristics of cohort of individuals with dementia, total and by each ethnic group. As ethnic group, Townsend score of social deprivation and comorbidities were imputed and sample sizes should not be reported for imputed data, only percentages are reported for characteristics of each ethnic group and for Townsend score

**Characteristic**	**Number (%**)	**%**	**%**	**%**
**Ethnic group**	**All**	**White**	**Asian**	**Black**
Individuals	20 821	96.6%	2.1%	1.2%
**Sex**				
Male	7457 (35.8%)	35.6%	42.5%	38.9%
**Age (years**)				
Mean (95% CI; SD)	79.79 (79.67 to 79.91; 9.03)	79.9 (79.78 to 80.03; 9.36)	77.27 (76.23 to 78.31; 8.94)	75.30 (73.79 to 76.80; 10.63)
**Age category, years**				
50–59	734 (3.5%)	3.4%	4.6%	10.3%
60–69	2231 (10.7%)	10.6%	14.5%	15.9%
70–79	6602 (31.7%)	31.4%	42.4%	39.7%
80–89	9091 (43.7%)	44.1%	33.4%	29.4%
≥90	2163 (10.4%)	10.6%	5.1%	4.8%
**Townsend quintile**				
1 (least deprived)	19.9%	20.1%	17.0%	12.1%
2	24.4%	24.7%	22.9%	11.5%
3	22.4%	22.3%	24.1%	21.3%
4	19.5%	19.4%	22.1%	24.9%
5 (most deprived)	13.8%	13.6%	13.9%	30.2%
**Prescribing index (95% CI; SD**)	8.29 (7.99 to 8.60; 2.28)	8.34 (8.02 to 8.66; 2.29)	9.09 (7.55 to 10.62; 2.33)	6.51 (4.67 to 8.35; 2.74)
**Comorbidity**				
Myocardial infarction	8.9%	8.9%	12.3%	5.1%
Stroke	22.7%	22.7%	23.6%	18.6%
Chronic kidney disease	43.6%	43.4%	49.6%	47.4%
Diabetes	59.3%	58.8%	72.8%	75%
Hypertension	55.4%	55.3%	61.0%	59.3%
No comorbidity	12.7%	12.8%	7.7%	8.8%

CI = confidence interval. SD = standard deviation.

The mean age was 79.8 years. Among this cohort, 59% had diabetes, 55% had hypertension, 44% had CKD, 23% had a stroke diagnosis, 9% had an MI diagnosis, and 13% had no comorbidity recorded.

There were some slight differences in age and sex distribution between those with and without ethnic group recorded (Supplementary Table 2).

### Annual dementia review

An annual dementia review was recorded for 14 105 (68%) individuals with dementia. There was no significant difference in the proportion of people with dementia from black, Asian, or white ethnic groups receiving a review (Table 2). Neither was there significant difference in the proportion of people with dementia receiving a review according to comorbidity status (Table 3).

**Table 2.** Incidence rate ratio and 95% confidence interval (CI) for each of the five health monitoring outcomes in the dementia cohort (*n* = 20 821) and ethnic and comorbidity status. Results are shown for unadjusted models and model adjusted for sex, age, Townsend score, prescribing index, and comorbidities

	**Dementia QOF review**	**BP monitoring**	**GP consultation**	**Weight/BMI**	**Influenza vaccination**
	**Unadjusted**	**Adjusted^a^**	**Unadjusted**	**Adjusted^a^**	**Unadjusted**	**Adjusted^a^**	**Unadjusted**	**Adjusted^a^**	**Unadjusted**	**Adjusted^a^**
**Total**	14 105(67.7%, 67.1% to 68.4%)		16 611(79.8%, 79.2% to 80.3%)		20 231(97.2%, 96.9% to 97.4%)		**9927** **(47.7%, 46.9% to 48.4%**)		**16 781** **(80.6%, 80.0% to 81.1%**)	
**Ethnic group**										
White	1	1	1	1	1	1	1	1	1	1
Asian	0.98(0.87 to 1.12)	1.03(0.91 to 1.17)	1.08(0.97 to 1.20)	1.04(0.93 to 1.16)	0.99(0.90 to 1.09)	0.98(0.89 to 1.08)	0.91(0.77 to 1.08)	0.84(0.71 to 1.00)	0.99(0.89 to 1.11)	0.99(0.88 to 1.11)
Black	0.88(0.74 to 1.06)	0.96(0.80 to 1.15)	0.93(0.80 to 1.09)	0.93(0.80 to 1.09)	0.95(0.83 to 1.08)	0.95(0.84 to 1.09)	0.83(0.65 to 1.06)	**0.77** **(0.60** **to 0.98**)	0.88(0.75 to 1.03)	0.92(0.78 to 1.10)
**≥1 comorbidity**	1	1	1	1	1	1	1	1	1	1
**No comorbidity**	1.05(1.00 to 1.10)	1.06(1.00 to 1.12)	0.69(0.65 to 0.73)	0.71(0.68 to 0.75)	0.97(0.93 to 1.01)	0.98(0.94 to 1.03)	**0.72** **(0.67** **to 0.77**)	**0.74** **(0.69** **to 0.79**)	0.92(0.88 to 0.97)	0.95(0.91 to 1.00)

BMI = body mass index. BP = blood pressure. QOF = Quality and Outcomes Famework.

^a^A full table of covariates used in the adjusted model can be found in Supplementary Table S3.

**Table 3.** Comorbidity characteristics of cohort for each of the five health monitoring outcomes (*n* = 20 821)

	**MI**(***n* = 1864**)	**Stroke**(***n* = 4717**)	**Diabetes**(***n* = 12 343**)	**CKD**(***n* = 9074**)	**Hypertension**(***n* = 11 542**)	**No comorbidity**(***n* = 2641**)
Dementia QOF review	1214 (65.1%)	3315 (70.3%)	8176 (66.2%)	6101 (67.2%)	7870 (68.2%)	1870 (70.8%)
BP monitoring	1660 (89.1%)	4106 (87.1%)	10 419 (84.4%)	7820 (86.2%)	10 101 (87.5%)	1513 (57.3%)
GP consultation	1826 (98.0%)	4645 (98.5%)	12 060 (97.7%)	8889 (98%)	11 299 (97.9%)	2502 (94.7%)
Weight/BMI	968 (51.9%)	2366 (50.2%)	6506 (52.7%)	4775 (52.6%)	5848 (50.7%)	938 (35.5%)
Influenza Vaccination	1601 (85.9%)	3991 (84.6%)	10 039 (81.3%)	7560 (83.3%)	9572 (82.9%)	1985 (75.2%)

CKD = chronic kidney disease. BMI = body mass index. BP = blood pressure. MI = myocardial infarction. QOF = Quality and Outcomes Framework.

### Annual blood pressure monitoring

At least one BP check was recorded in 16 611 (80%) individuals with dementia, with an average of 2.9 BP recordings per person per year. There was no significant difference in the proportion of people with dementia from black, Asian, or white ethnic groups receiving at least one BP check. Those without comorbidities were less likely to have at least one BP check, compared with those with at least one comorbidity (78% versus 82%; adjusted IRR = 0.95, 95% CI = 0.92 to 0.98) (Table 2).

### Number of GP consultations

At least one GP surgery, telephone or home visit consultation was recorded in 20 231 (97%) individuals with dementia. There was no significant difference in the proportion of people with dementia from black, Asian, or white ethnic groups receiving at least one GP consultation. There was also no significant difference in the proportion of people with dementia according to comorbidity status receiving at least one GP consultation (Table 2).

### Annual weight and/or BMI recording

A weight and/or BMI was recorded in 9927 (48%) individuals with dementia. Compared with white groups, black people were 23% less likely (adjusted IRR = 0.77, 95% CI = 0.60 to 0.98) and Asian people 16% less likely (adjusted IRR = 0.84, 95% CI = 0.71 to 1.00) to have their weight recorded. People with dementia without any comorbidities were less likely to have at least one weight and/or BMI check within a year, compared with people with at least one comorbidity (36% versus 49%; adjusted IRR = 0.74, 95% CI = 0.69 to 0.79) (Table 2).

### Annual influenza vaccination

Receipt of the annual influenza vaccination was recorded in 16 781 (81%) individuals with dementia. There was no significant difference in proportions of people with dementia from black, Asian, or white ethnic groups receiving the vaccination. There was also no significant difference in the proportion of people with dementia according to comorbidity status receiving the vaccination (Table 2).

## Discussion

### Summary

In this large UK primary care study, only two-thirds of people with dementia received an annual review, despite guidance recommending this as a minimum standard.^7^ Less than half of people with dementia in the study had a weight and/or BMI recorded; this happened less frequently in people from black and Asian ethinic groups compared with white ethnic groups, and less frequently in people without comorbidities compared with those with ≥1 comorbidity. A fifth of people with dementia did not receive an influenza vaccine or a BP check within a year, and only 57% of people with dementia without other comorbidities had a BP check. Ethnic group was not associated with differences in BP monitoring, GP consultations, influenza vaccination, or dementia annual review.

### Strengths and limitations

THIN comprises around 6% of the UK population, and is broadly representative in terms of demographic and health variables.^13^ Ethnic group was imputed to address missing data, and results from complete case and multiple imputation analyses were similar (Supplementary Table 1). Imputing missing data meant the full sample of individuals with dementia could be used to provide more precise estimates of outcomes.

People living with dementia were identified through a coded dementia diagnosis or anti-dementia drug repeat prescription, which may exclude a small number diagnosed with dementia within secondary care and not captured in GP records.

The study focused on ethnic group as black, Asian, or white as data at more detailed levels were not well recorded. However, this may have obscured smaller intra-group differences. Ethnic groups are considered to share a common ancestry, culture, and feeling of solidarity with one another.^22^ There is wide variation within minority ethnic groups in country of origin, language, religion, socioeconomic characteristics, and experiences, but enough shared culture with regards to family structures, identity, and health beliefs to make ethnic group relevant to health behaviours.^22^ While some comorbidities were accounted for, analyses may have been confounded by unmeasured variables (for example, overall physical health, frailty, social demographics) affecting service use. This study also had relatively low numbers of people from black and Asian backgrounds, which may have obscured smaller differences between groups.

The study investigated the likelihood of receiving at least one BP or weight and/or BMI measurement, which is a crude binary outcome and it is not known whether those with abnormal values received adequate treatment or follow-up. While Read codes were used to capture influenza vaccination delivered in other settings, some may have been missed if they were not documented in GP records.

### Comparison with existing literature

To the authors' knowledge, this is the first study to explore routine GP physical health monitoring and ethnic group in people with dementia. The finding that black and Asian ethnic groups are less likely to have their weight and/or BMI recordedx adds to other known barriers to good dementia care for these groups.^9,10^ Contextual barriers to help-seeking among these groups include negative experiences of health services and perceptions that caring is a family responsibility.^22–24^ However, for most outcomes, no differences between ethnic groups were found. This may indicate that among people with dementia, barriers to accessing physical health care are shared among all ethnic groups, or that smaller differences between groups were undetected owing to small numbers of minority groups. The study found weight and/or BMI had the lowest rates of completion of all the component of monitoring studied, so perhaps inequalities emerge in less routine care activities, because clinicians are more vulnerable to unconscious bias when exercising more discretion. Lower levels of overall comorbidity were identified among white groups compared with black and Asian groups, as is consistent with the literature.^9,11^

The findings contrast with those investigating mental health care, where minority ethnic groups are less likely to receive anti-dementia medication where potentially indicated and receive antipsychotic medication for longer.^10^ The impact of stigma on mental health care access compared with physical health care may explain this difference.

Between 2002–2013, dementia annual review uptake was under 50%.^25^ The results of the present study show an improvement in 2015–2016 (possibly related to QOF guidance),^7^ but still a third of people with dementia had no review recorded, despite most having at least one annual GP contact. This lack of review is important as pressures on secondary mental health services are rising, and responsibility for routine monitoring may increasingly fall to primary care. Guidelines for the annual dementia review are extensive; they emphasise a tailored review to consider physical health, mental health, nutrition, optimising polypharmacy, care needs, functional ability, and end-of-life discussions, among other areas.^7^ Such broad guidance may challenge GPs facing workload and time pressures.^26^ Some studies suggest that GPs may believe they have little to offer patients with dementia.^27^ The rationale for the dementia review needs to be clearly explained, including the importance of routine physical health monitoring to dementia outcomes. Other factors explaining low uptake may include varying QOF renumeration^28^ and stigma related to dementia;^29^ GPs may be undertaking the dementia review tasks, but not recording them as such.

While dementia detection and diagnosis rates in primary care are rising,^30^ the findings suggest optimal care post-diagnosis lags behind. The study found less than half of people with dementia had a weight and/or BMI recorded, consistent with another THIN study reporting 47% had this recorded in 2010–2013,^25^ and other reports of poor weight recording in primary care.^31^ Eating and drinking difficulties are common in dementia. The risk of malnutrition increases as dementia progresses and people with dementia have 10 times more malnutrition or dehydration-related hospital admissions compared with age-matched controls.^32^ Anti-dementia drugs causing side effects, such as nausea, can also exacerbate difficulties. Other research has highlighted concerns that nutrition support for older people is insufficient^33^ and weight loss may not be recognised as a problem.^34^

The study found over 80% of people with dementia had at least one BP check, one GP contact, and the influenza vaccination, confirming previous studies.^25,35^ An annual BP check is not specified in guidelines, although this was used as a marker of good preventive health care. While some studies suggest that good BP control can reduce risk of further cerebrovascular changes, others describe a complex relationship between optimal BP and dementia.^36^ People with dementia are more likely to experience postural hypotension and adverse effects of low BP,^37^ which is an argument for regular monitoring, regardless of comorbidities. However, standard BP monitoring may cause anxiety,^38^ so should be considered carefully.

### Implications for research and practice

The results suggest routine physical health monitoring for people living with dementia, particularly nutrition monitoring, is inconsistent and requires improvement. Much emphasis in primary care has been on dementia detection, where meaningful improvements have been achieved and sustained. Priorities should now shift to post-diagnosis support.^39^ Dementia should be considered a long-term health condition requiring routine monitoring in its own right. At present, guidelines for the annual dementia review are extensive. Prioritising components of the review that are realistic and achievable in general practice should be central to any revisions. Improved weight recording and management of nutrition should be prioritised, promoting ethnic and sex equalities in access. The role of BP monitoring, particularly for people living with dementia without other cardiovascular risk factors, should be considered by researchers. Defining best practice in physical healthcare monitoring and management of comorbidities could shape future dementia guidelines.
